# Synthesis of Co/Co_3_O_4_ Heterostructure in N-Doped Porous, Amorphous Carbon: A Superior Electrochemical Sensor for Sensitive Determination of Alectinib in Various Fluids

**DOI:** 10.1021/acsomega.4c04821

**Published:** 2024-10-24

**Authors:** Nesrin Bugday, Edoh Nicodème Gabiam, Nevin Erk, M.Soner Bay, Asena Ayşe Genc, Ozgur Duygulu, Sedat Yaşar

**Affiliations:** † Faculty of Science and Art, Department of Chemistry, İnönü Üniversity, Malatya 44280, Turkey; ‡ Faculty of Pharmacy, Department of Analytical Chemistry, Ankara University, Ankara 06100, Turkey; § TUBITAK Marmara Research Center, Materials Technologies, Gebze, Kocaeli 41470, Turkey

## Abstract

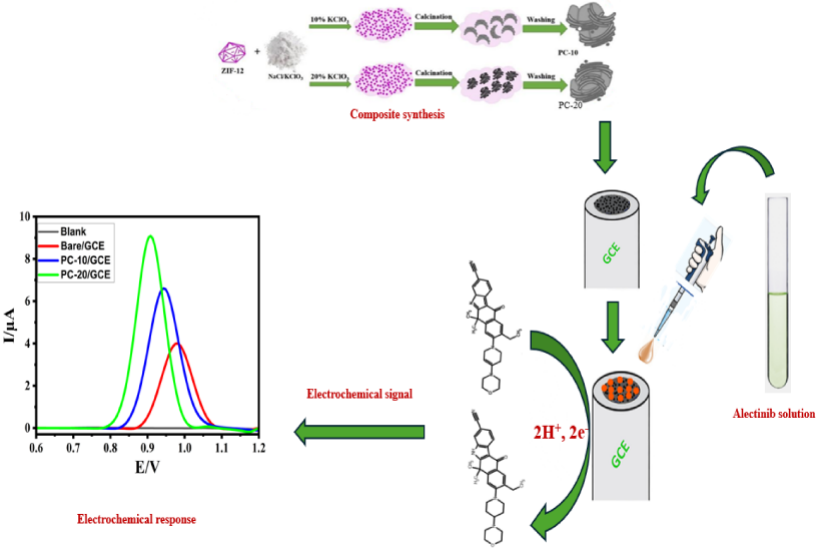

Highly crystallized Co and Co_3_O_4_ nanoparticles embedded in an N-doped amorphous carbon matrix have been successfully fabricated by the molten-salt-assisted method using KClO_3_ and zeolitic imidazolate framework-12 (ZIF-12). Pyrolysis of ZIF-12 with different concentrations of KClO_3_ leads to embedded Co and Co_3_O_4_ nanoparticles in a conductive amorphous carbon network. The impact of salt concentration on the morphology and electrochemical performance of these composites was investigated for electrochemical sensor applications. By employing a straightforward and efficient technique, Co/Co_3_O_4_ heterostructures were successfully synthesized in N-doped porous amorphous carbon. The Co/Co_3_O_4_ carbon heterostructures were optimized by varying the salt concentration, resulting in a significant electrochemical sensor performance for detecting ALC in both bulk and biological fluids. The sensor demonstrates excellent sensitivity (62.97 nmol/L) and selectivity toward ALC, with a wide linear range (0.2–2 μM) and a low detection limit (18.89 nM). Furthermore, it displays remarkable stability and reproducibility, positioning it as a strong contender for practical use in pharmaceutical analysis and biomedical research. This study presents a significant advancement in electrochemical sensing technology and underscores the potential of Co/Co_3_O_4_ heterostructures in the development of high-performance sensors for detecting bioactive compounds in complex matrices.

## 1 Introduction

The adaptable porosity structure and large surface area of metal–organic frameworks (MOFs) increase their potential for use in a variety of fields.^1^ Extended 3D network (RHO) zeolitic imidazolate framework-12 (ZIF12), which is composed of tetrahedrally coordinated cobalt ions and four nitrogen atoms of benzimidazole (bIm), has gained significant attention for its potential in various applications as an electroactive material. In order to augment the practical utility of carbons obtained from MOFs across several domains, recent research has been dedicated to the synthesis of hierarchical porous carbons from MOFs.^2^ Several ZIF designs for the production of extremely efficient electroactive materials have been presented.^3−7^ However, no report exists on the fabrication of carbon and cobalt nanoparticles/cobalt oxide composites employing a single ZIF-12 as the electroactive material for an electrochemical sensor. The carbonization of ZIF-12 under an argon atmosphere creates N-doped porous carbon materials and electroactive cobalt nanoparticles/Co_3_O_4_ heterostructures. For the storage of charges, the combination of N-doped porous carbon material and Co_3_O_4_ as active materials for energy storage devices is quite promising. With this approach, Co/Co_3_O_4_ embedded in an N-doped porous carbon material could play a significant role as electrochemical sensors in drug transport systems.

The majority of previous research on carbons derived from MOFs focused on the direct pyrolysis.^8−10^ Salting-templating^11^ has recently been identified as a straightforward and environmentally friendly technique. When a carbon source is mixed with an inorganic salt and exposed to high pyrolysis temperatures, the mixture transforms into a carbonized structure. This process produces carbonized networks that preserve the framework of their inorganic equivalents while retaining their outstanding porosity and pore size. Producing high quantities of carbon-based materials with little effort and expense is possible through molten salt synthesis.^12−16^ This method is commonly used to produce different carbon nanostructures utilizing different carbon precursors and inorganic salts.^17−23^

ZIF-12 was modified for the first time in this study by adjusting the quantity of KClO_3_, the carbonization stage, and the decorating of Co/Co_3_O_4_ embedded in N-doped porous carbon. The corresponding carbonization operations were conducted under argon flow at 800 °C. By carbonizing ZIF-12/NaCl–KClO_3_ (cobalt, benzimidazole, NaCl, and KClO_3_) in a single pot, cobalt nanoparticles, Co_3_O_4_, and N-doped porous carbon are produced. A proposed investigation outlines the intricate design process and potential composition of cobalt nanoparticle/Co_3_O_4_/N-doped porous carbon derived from ZIF-12/NaCl-KClO_3_, aiming to elucidate the electrochemical sensing capabilities of the synthesized active materials under varying NaCl–KClO_3_ ratios. Among the derivatives produced through sequential carbonization steps, the PC-20 variant exhibited the most superior electrochemical sensing capability, attributed to the orderly configuration of electroactive cobalt nanoparticle/Co_3_O_4_/N-doped porous carbon within its structure. The amorphous carbon sheets (NCSs) are N-doped and have many defects. The 3D shape promotes mass diffusion, while the nanosheets facilitate electrical conductivity. Following the elimination of salt, the interconnected carbon skeletons that are produced transform into macropores, facilitating rapid electron–ion transport channels. This study introduces novel design principles for cobalt nanoparticle/Co_3_O_4_/N-doped porous carbon obtained from ZIF-12 using straightforward calcination procedures and pure ZIF12/NaCl-KClO_3_ materials.

**Figure 1:**
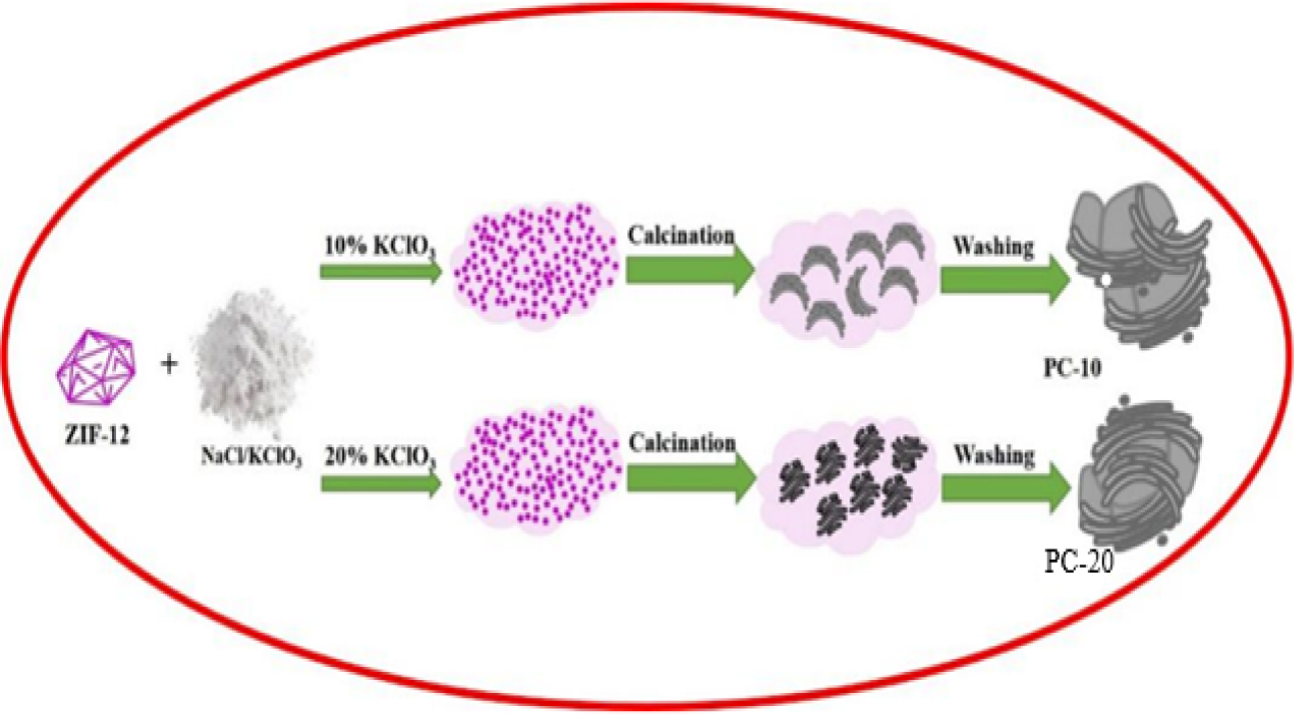
Synthesis processes for PC materials are illustrated in brief.

Alectinib (ALC), chemically known as (9-ethyl-6,6-dimethyl-8-[4-(morpholin-4-yl)piperidin-1-yl]-11-oxo-6,11-dihydro-5*H*-benzo[b]carbazole-3-carbonitrile hydrochloride (Figure 1), is a medication designed to target and inhibit the activity of anaplastic lymphoma kinase (ALK) within the central nervous system.^24^ ALC is recognized as a highly effective second-generation blocker of ALK, displaying strong efficacy against various mutations of ALK, and has been found to effectively target and inhibit ALK mutations across a broad spectrum.

The US Food and Drug Administration (FDA) and the European Medicines Agency (EMA) have both granted approval for the use of ALC as a first-line treatment in patients with ALK-positive metastatic nonsmall cell lung cancer.^25^

The ALK gene rearrangement leads to the expression of an abnormal ALK protein, which plays a role in the uncontrolled growth of cancer cells.^26^ ALC works by specifically targeting and inhibiting the activity of this abnormal ALK protein. By doing so, it helps to block the signaling pathways that promote the growth and survival of cancer cells, ultimately slowing or inhibiting the progression of the cancer.

Numerous analytical methods have been documented in the literature for the analysis of ALC in different sample matrices. These methods include HPLC-FD,^27^ LC-MS/MS,^28^ and UPLC-MS/MS.^29^

When compared to HPLC, HPLC-MS is notably more acute, precise, and effective for both identifying and quantifying ALC. However, the instrumentation required for LC-MS analysis is costly and may not be accessible in numerous laboratories. The HPLC method is extensively utilized in bioavailability studies because of its affordability and superior durability. However, HPLC assays are often hindered by issues related to sensitivity and specificity, which limits their effectiveness in certain analytical contexts. Due to the low concentrations of ALC present in real samples, there is a critical need to develop a fast, eco-friendly, and accurate chemical technique for its detection across various matrices. Despite an extensive review of the existing literature, no electrochemical method for the determination of ALC was found. Consequently, our study presents a pioneering electrochemical approach for the determination and quantification of ALC, utilizing the distinctive characteristics of a newly developed PC-20 composite. The PC-20 composite, developed through a meticulous combination of synergistic materials, demonstrates outstanding electrochemical characteristics that improve the precision and accuracy of detection of ALC. This innovation has the potential to revolutionize precision in cancer therapy by offering clinicians a reliable and efficient tool for monitoring drug concentrations in patient samples. As we explore the details of this electrochemical strategy, our objective is to clarify its superiority compared to traditional approaches and emphasize its revolutionary impact on the field of oncology.

## 2 Material and Methods

### 2.1 Chemicals

To access additional details and information, please refer to the Supporting Information.

### 2.2 Synthesis of ZIF-12

The synthesis of ZIF-12 was synthesized as in our previous study.^30^

### 2.3 Preparation of Porous Carbon (PCs)

In a typical synthesis, 1 g of prepared ZIF-12 was combined with 5 g of a highly concentrated salt solution, with ratios of NaCl/KClO_3_ at either 9:1 or 8:2 by weight. This mixture underwent vigorous stirring for more than 24 h until the ZIF-12 precursor was surrounded by the salt solution. The temperature was elevated to 60 °C until complete recrystallization of the salt occurred. The final product was dried under high vacuum for 20 h. Then, the ZIF-12@NaCl/KClO_3_ was heated at 800 °C for 3 h under the heating rate of 2 °C per minute. Upon reaching room temperature, the product was obtained by rinsing with distilled water and then filtering and subsequently drying at 120 °C. The products are known as PC-10 and PC-20, where 10 and 20 represent the weight ratio of used KClO_3_. The synthesis steps are listed in Figure 1.

### 2.4 Fabrication of Modified Electrodes

In this study, two different electrodes modified by PC-10 and PC-20 were fabricated by utilizing liquid-phase coating (wet process). Initially, the pristine glassy carbon electrode (GCE) underwent polishing with an alumina powder. Next, the GCE underwent a sonification process in a bath containing methanol and deionized (DI) water in a 1:1 ratio to eliminate potentially absorbed particles. Following this, the cleansed GCE surface was washed with distilled water and then underwent air-drying for further use. Two different solutions were prepared by dissolving 1 mg of each composite (PC-10 and PC-20) in 1 mL of distilled water, accompanied by 1 h of ultrasonic treatment. Subsequently, 6 μL of the resulting dispersion was introduced into an electrochemical cell, which already contained 9 mL of the Britton–Robinson (BR) solution.

The pretreated bare electrode was submerged in a mixture solution of Britton–Robinson buffer and composite. Furthermore, it underwent a coating process at a constant potential for a duration of 100 s.

### 2.5 Real Samples

To analyze ALC content in tablets, 10 tablets of ALC, each containing 600 mg, were crushed into powder; 10 g of the obtained powder was dissolved in a 100 mL volumetric flask using ultrasonication.

The urine sample obtained from a healthy volunteer was filtered utilizing a 0.45 μm PTFE syringe filter. Then, 5 mL of the filtered sample was rigorously mixed with 5 mL of ALC.

The determination of ALC in real samples was conducted according to a standard addition method. This method entailed supplementing the samples with known amounts of ALC and analyzing the resultant solutions using predetermined analytical parameters. Following this, the concentration of ALC in the samples was deduced by evaluating fluctuations in the electrochemical signals, enabling accurate quantification within the real samples.

## 3 Results and Discussion

### 3.1 PCs Nanocomposites Characterization

**Figure 2:**
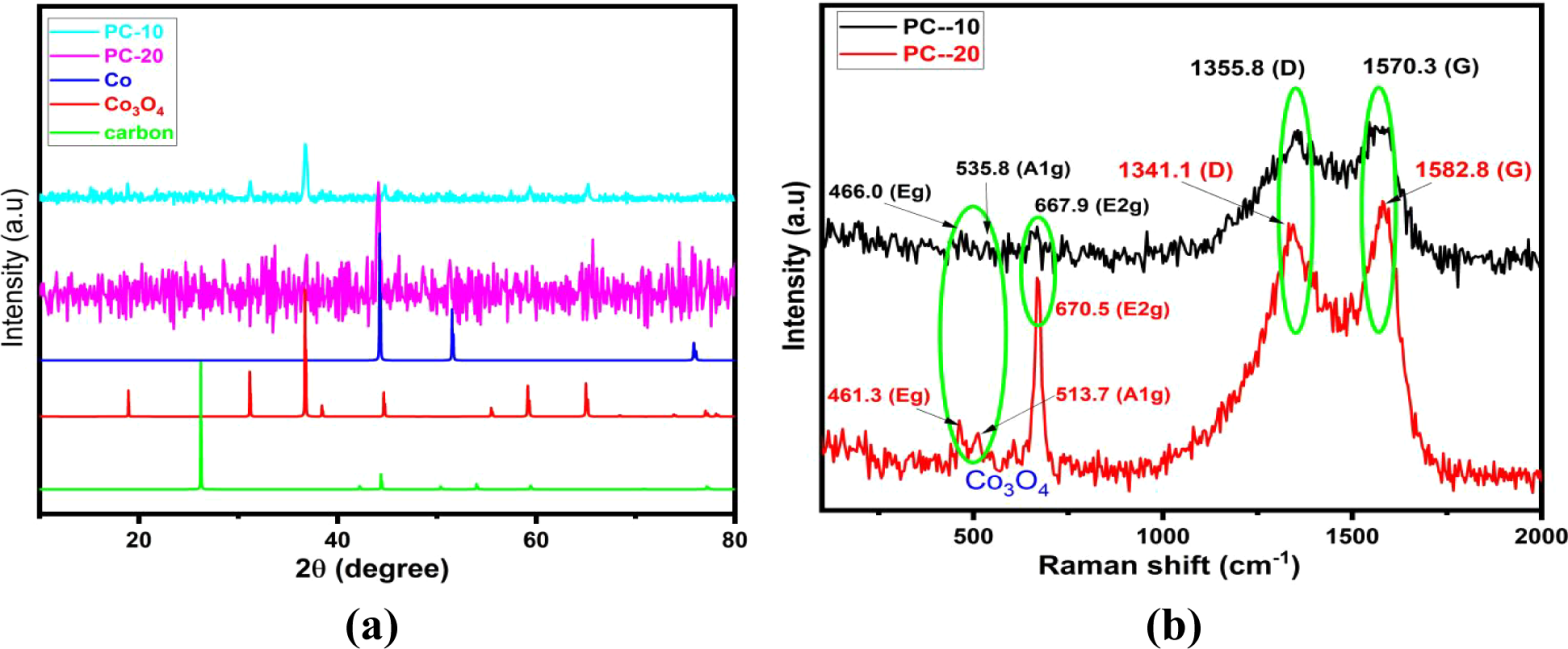
XRD (a) and Raman (b) analysis of PC-10 and PC-20.

Structural characterization of PC-10 and PC-20 nanocomposite materials started with X-ray diffraction (XRD). The XRD patterns of the PC-10 and PC-20 materials are given in Figure 2a. According to XRD analysis, the PC-10 material was mainly composed of amorphous carbon with Co_3_O_4_, while the PC-20 material was composed of amorphous carbon, metallic Co nanoparticles and Co_3_O_4_. While the PC-20 sample matches well with the metallic Co (PDF: 15-0806), the PC-10 sample matches well with the Co_3_O_4_ crystals (PDF: 43-1003), which indicates that the formation of metallic Co and Co_3_O_4_ crystals can be mainly attributed to the concentration of KClO_3_ salt. No sharp peak is observed at 26.60, indicating that the carbon in the structure of PC-10 and PC-20 is close to an amorphous structure. This is also supported by the SEM analyses. However, carbon content in PC materials can be evaluated by using the SEM-EDS analysis. As shown in Table S1, the carbon content of the PC-10 and PC-20 was 62.14% and 35.78%, respectively.

The XRD and SED-EDS analysis shows that ZIF-12 in the molten salt medium has been transformed to mainly Co/Co_3_O_4_ crystals by pyrolysis, resulting in the formation of the Co/Co_3_O_4_/PC materials. However, According to XRD analysis of PC materials, PC-10 and PC-20 consist of mainly Co_3_O_4_, amorphous carbon, and a tiny amount of metallic Co nanoparticles. The above result indicates that KClO_3_ salt contains oxygen in its structure contribute to the formation of Co_3_O_4_ even calcination process realizes under argon atmosphere. Furthermore, the observation is confirmed by Raman analysis, as shown in Figure 2b. Figure 2b displays the Raman spectra of the PC samples. For PC-10, the peaks at 466.0, 535.8, and 667.9 cm^–1^ correspond to *E*_g_, *F*_22g_, and *A*_1g_ modes of vibration, respectively.^31^ In the case of the PC-20 sample, vibrations are detected at 461.3, 513.7, and 670.5 cm^–1^, consecutively. Bulk Co_3_O_4_ exhibits Raman peaks at 482.4, 521.6, and 691 cm^–1^, respectively.^32^ The 665 cm^–1^ band noted in PC-10 and PC-20 corresponds to the symmetric stretching vibration of the octahedral (CoO_6_) group, classified as the *A*_1g_ species within the *O*_h7_ spectroscopic symmetry. The *E*_g_ and *F*_22g_ are combined vibrational modes of the tetrahedral site and octahedral oxygen in the Co_3_O_4_ system.^33^ The presence of these peaks provides additional evidence that both PC-10 and PC-20 contain Co_3_O_4_ in spinel structures.^34^ It is evident that all of the peaks in PC-10/PC-20/Co_3_O_4_ exhibit a red shift compared to bulk Co_3_O_4_, which can be attributed to the smaller particle size and the presence of oxygen defects. The defect (D) and graphitize (G) bands of the PC-10 and PC-20 materials reveals at slightly different shifts with different intensities. The *ID*/*IG* ratio for the PC-10 and PC-20 was calculated as 0.863 and 0.847. However, the observation of *E*_g_, *A*_1g_, and *E*_2g_ bands of Co_3_O_4_ in the Raman spectra confirms the formation of Co_3_O_4_ in the PC-10 and PC-20 materials.

**Figure 3:**
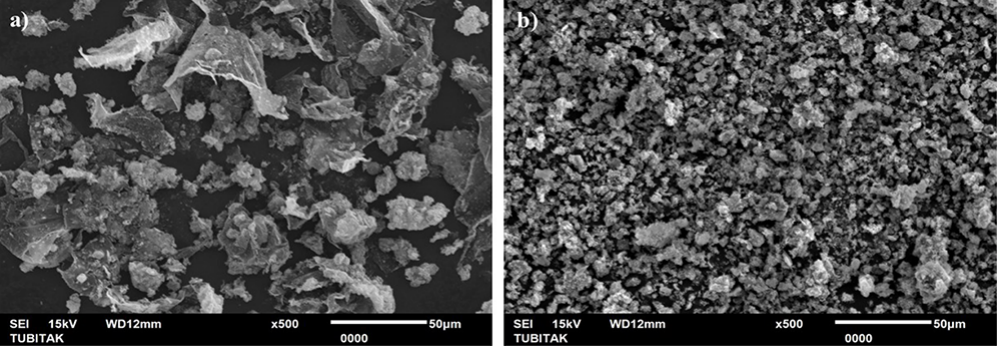
SEM images of the PC-10 (a) and PC-20 (b) specimens.

The scanning electron microscopy (SEM) images of PC-10 and PC-20 specimens are given in Figure 3a,b, respectively. The PC-10 sample has been found to have 2D carbon structures that resemble graphene. The PC-20 sample has a more homogeneous morphology, with less aggregation than the PC-10 sample.

**Figure 4:**
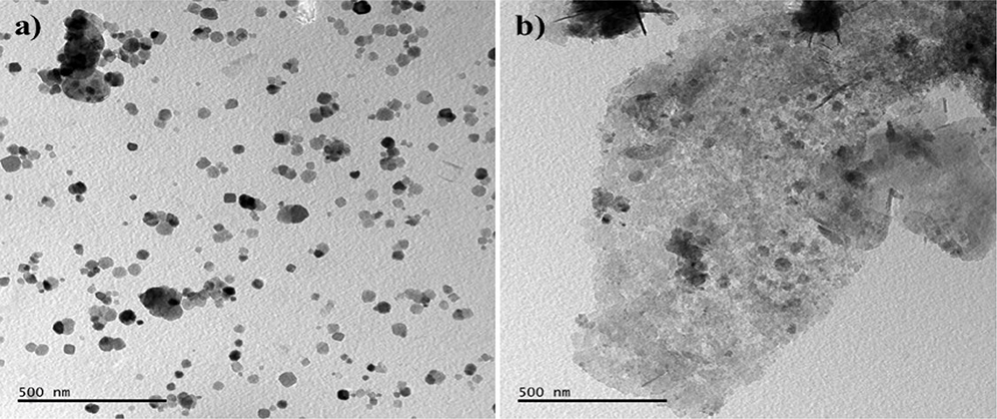
TEM images of PC-10 (a) and PC-20 (b) samples.

**Figure 5:**
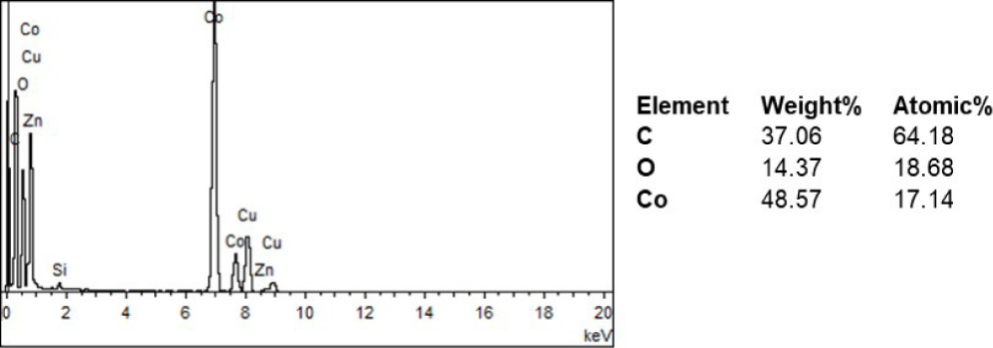
TEM-EDS spectrum and result of Co_3_O_4_ nanoparticle.

**Figure 6:**
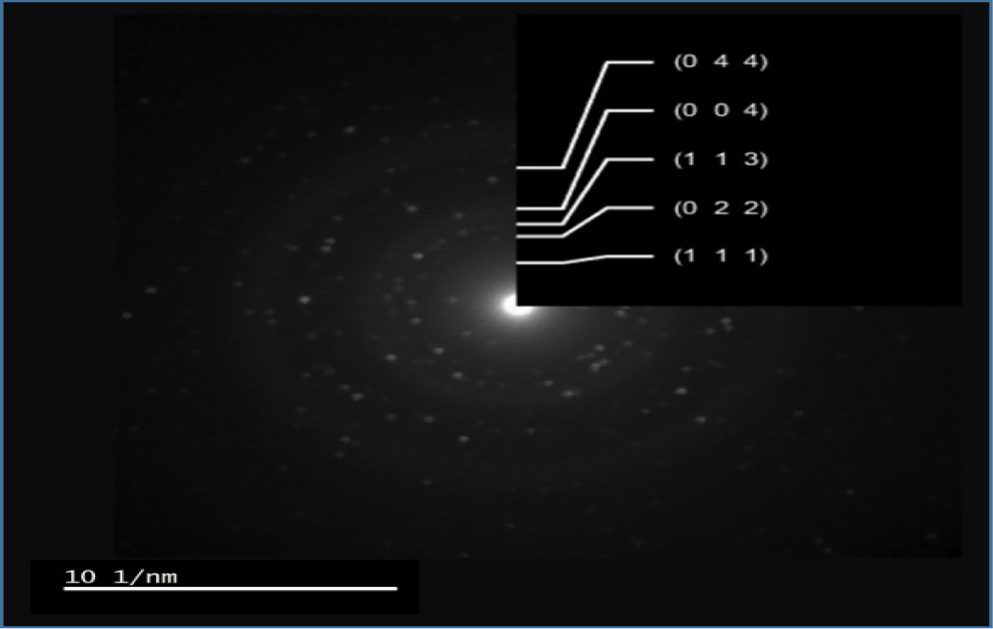
Selected area electron diffraction (SAED) pattern and indexation results of Co_3_O_4_ nanoparticles.

In Figure 4, TEM images of the PC-10 (a) and PC-20 (b) samples are shown. Thin amorphous carbon layer matrix of PC-10 is more obvious. It is also evident that the nanoparticle sizes of the two specimens are comparable. With the help of TEM-EDS it can be stated that the majority of the nanoparticles are Co_3_O_4_ (Figure 5). TEM-SAED (selected area electron diffraction) pattern indexation also matches with the major (hkl) planes of Co_3_O_4_ phase as shown in Figure 6

**Figure 7:**
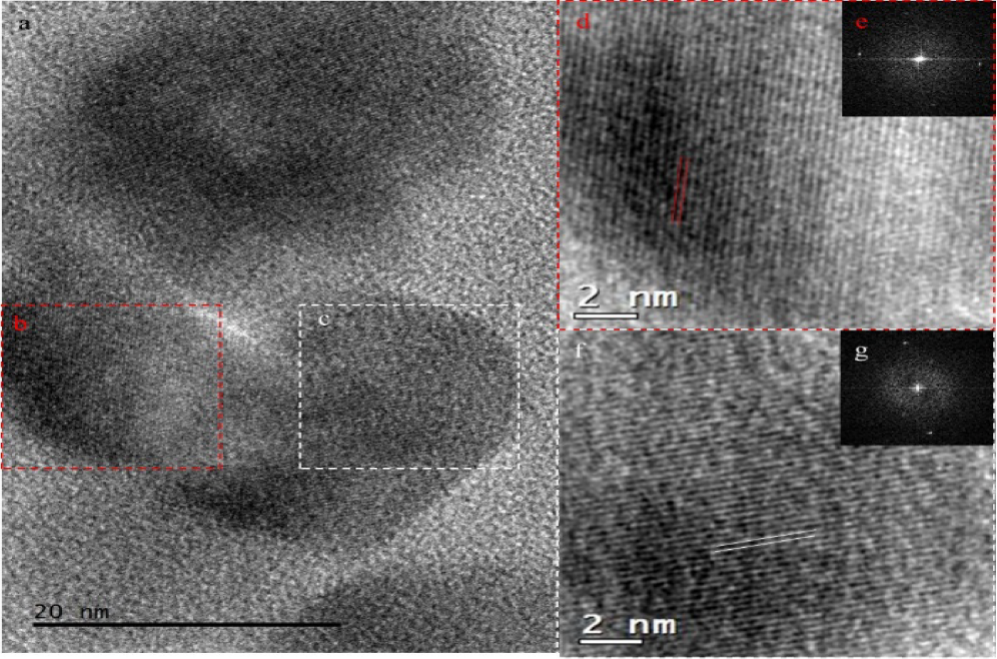
(a) HRTEM image and (e,g) FFT diffractograms of the PC-10 sample. HRTEM images labeled (d) and (f) are magnified views of (b) and (c), respectively, showing (220) *d*-spacings of Co_3_O_4_.

HRTEM and fast Fourier transformation (FFT) diffractograms investigations for PC-10 also helped to understand the size, shape, and phase of nanoparticles. In Figure 7a, representative HRTEM study is shown for Co3O4 nanoparticles. HRTEM image sample. HRTEM images labeled (d) and (f) are magnified views of (b) and (c), respectively, showing (220) *d*-spacings of Co3O4.

**Figure 8:**
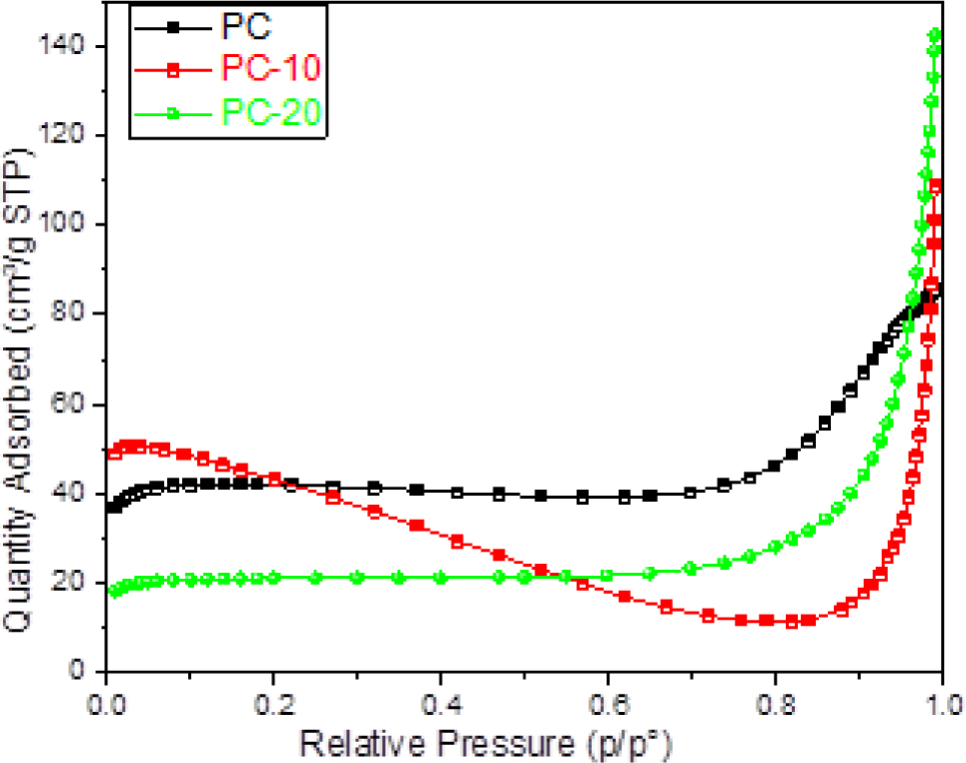
Nitrogen adsorption isotherms of PC, PC-10, and PC-20.

The nitrogen gas absorption curves of PC-10 and PC-20 (Figure 8) suggest that the specific surface area of the PCs was slightly enhanced. Table 1 provides a specific listing of the BET surface areas of the final products and the precursor. PC-10 was observed to have the greatest surface area, as shown in Table 1. It could be the result of ZIF-12’s structural collapse, which was induced by the high KClO3 concentration. BET analyses, which are consistent with SEM and TEM pictures, demonstrated that the PCs had a microporous structure, in particular. Evaluation of the adsorption diagrams reveals that all samples contain Type 1 isotherms, confirming the microporous pore structure of NPCs.

**Table 1:** TEM EDS of PC, PC-10 and PC-20

Compound	C%	Co%	BET Surface Area (m^2^/g)
PC-10	74.70	25.30	119. 21
PC-20	91.37	8.63	63.53
PC	67.55	32.45	128.6

**Figure 9:**
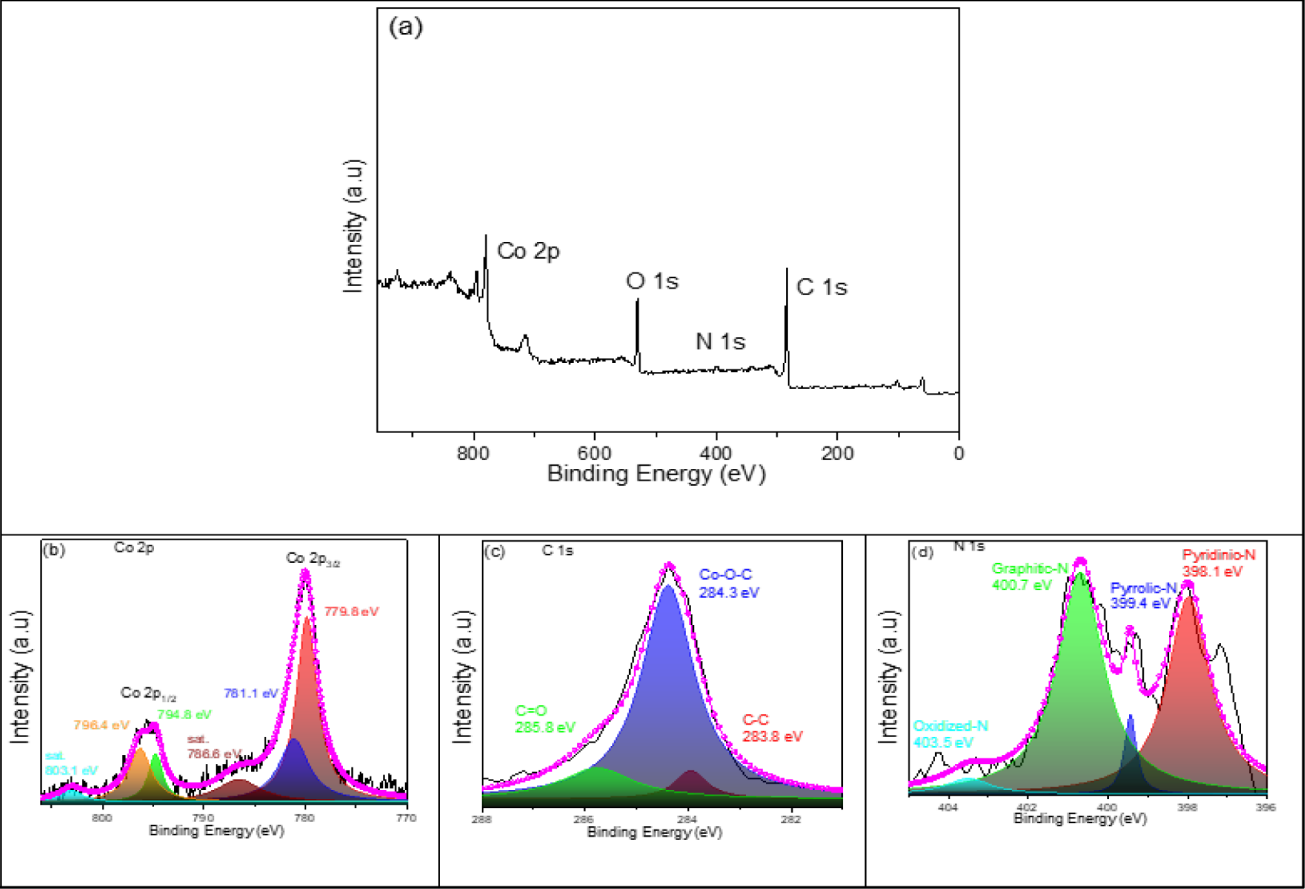
Survey XPS spectra of PC-20 (a) and high-resolution XPS spectra of Co 2*p* (b), C 1*s* (c), and N 1*s* (d).

Figure 9 shows the raw and fitted XPS analysis for the PC-20 material. The Co 2*p*, O 1*s*, N 1*s*, and C 1*s* peaks are located at 780.1, 530.3, 399.6, and 284.3 eV, respectively, as shown in Figure 9a–d. The high-resolution Co 2*p* XPS spectrum has four main peaks at 779.8, 781.1, 794.8, and 796.4 eV and two satalite peaks at 788.8 and 803.1 eV, respectively. Peaks that appear at 779.8 and 781.1 eV can be assigned to the Co 2*p*_1/2_, and peaks that appear at 794.8 and 796.4 eV can be assigned to the Co 2*p*_3/2_ of Co_3_O_4_, demonstrating that Co has Co^2+^ and Co^3+^ oxidation states.^35,36^ As can be calculated from XPS result, the content of carbon in the PC-20 is approximately 41.18 wt%. The peaks at 283.8, 284.3, and 285.8 eV can be assigned to C–C, Co–O–C, and C=O, respectively. The high-resolution N 1*s* spectrum reveals that PC-20 contains pyridinic-N (398.1 eV), pyrrolic-N (399.4 eV), graphitic-N (400.7 eV) and oxidized-N (403.5 eV). High contents of pyridinic-N and graphitic-N can promote the transfer of electrons throughout the composite carbon network of PC-20. The content of N element in NPCs is about 1.10% according to XPS analysis.

### 3.2 Evaluation of the Analytical Capabilities of the PC-10/GCE and PC-20/GCE

**Figure 10:**
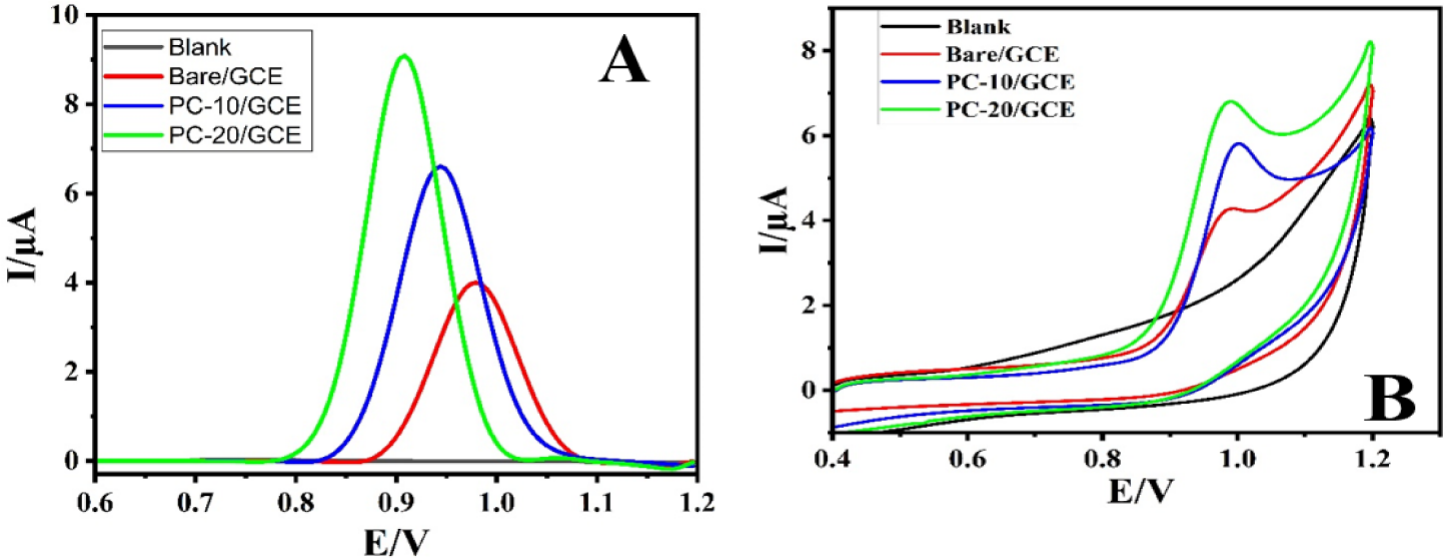
DPV (A) and CV (B) curves of 0.1 mM ALC in BR obtained at the surface of pristine GCE, PC-10/GCE, and PC-20/GCE.

The investigation into the efficacy of the two different synthesized composites (PC-10 and PC-20) for the ALC determination was realized through differential pulse voltammetry (DPV) and cyclic voltammetry (CV). First, a comprehensive investigation was carried out to study the electrochemical characteristics of ALC on both bare electrodes and electrodes modified with composites. The absence of an oxidation peak on the modified electrodes in the absence of ALC (blank) confirmed the inert characteristics of the utilized composites. Upon introducing 0.1 mM ALC, the bare GCE exhibited an oxidation peak with a current of 4.05 μA. The comparative analysis between the two composites revealed distinct differences in their electrochemical response toward ALC. The composite with PC-20 exhibited higher current response of 9.16 and lower peak potential compared to PC-10 in both DPV and CV measurements as shown on Figure 10A,B. This superior performance can be attributed to factors, such as the composition, morphology, and surface properties of the composites. Consequently, PC-20 was selected as the appropriate composite for the termination of ALC; thus, it was selected for the entire experimental procedure.

**Figure 11:**
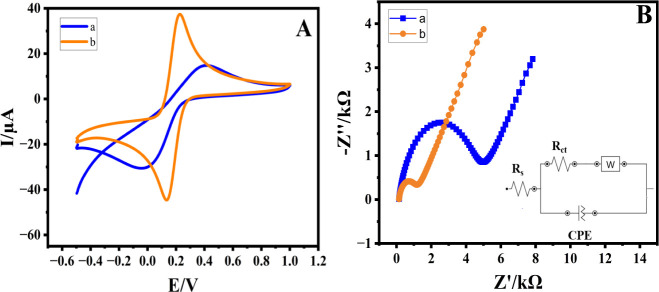
CV at scan rate of 50 mV s^–1^ (A) and EIS (B) curves of 5 mM K_4_[Fe(CN)_6_]/K_3_[Fe(CN)_6_] in 0.1 M KCl obtained at the surface of pristine GCE (a) and PC-20/GCE (b).

To confirm the electrocatalytic performance of the PC-20 nanoplate-modified glassy carbon electrode (PC-20/GCE), as depicted in Figure 11a, and the redox couple system of 5 mM K_4_[Fe(CN)_6_]/K_3_[Fe(CN)_6_] in 0.1 M KCl was chosen, and CV and electrochemical impedance spectroscopy were conducted. At the bare GCE, cyclic voltammetry displayed the lowest current density (curve a). Introducing a developed PC-20 nanocomposite onto the bare electrode resulted in a higher current density (curve b) attributed to diminished electron transfer resistance.

At a scan rate of 50 mV/s, the potential peak separations were determined to be 87.89 and 141.6 mV for PC-20/GCE and unmodified GCE, respectively. These findings provide additional validation for the enhanced electrochemical performance attained by integrating PC-20 nanocomposites onto the surface of the GCE.

Significantly higher electron transfer resistance (RCT) was evident at the pristine electrode (5289 Ω) (a), whereas upon application of the PC-20 nanocomposite onto the GCE surface (1203 Ω) (b), RCT notably decreased. This reduction underscores the outstanding electronic conductivity of PC-20.

The surface areas of both the pristine electrode and electrode-modified PC-20 were assessed utilizing the Randles–Sevcik equation (eq S1) and slope analysis of the square root of the scan rate versus the peak current (Figure S1.). The results show that the electroactive surface area was determined to be 0.088 cm^2^ for the bare GCE and 0.153 cm^2^ for the GCE modified with PC-20. These findings highlight that the developed PC-20 demonstrates the highest electroactive surface area, consequently offering a larger number of reactive sites.

### 3.3 Optimization of the Method

The optimization process focused on several parameters: choice of supporting electrolyte, pH influence, volume, and concentration of PC-20 nanocomposite. To commence this process, the initial step involved selecting the most suitable buffer solution. Various buffers, including potassium chloride, sodium hydroxide, Britton–Robinson (BR), phosphate buffer saline (PBS), potassium chloride, hydrochloric acid, and acetate buffer underwent evaluation utilizing the DPV method with ALC (0.1 mM). Among these options, BR buffer at pH 2.0 exhibited optimal response in terms of peak current, thus it was selected for the complete experimental procedure (Figure S2A). Following this, the impact of varying concentrations of the PC-20 composite was investigated across a range of 0.1–2.0 mg/mL. The highest peak current was detected at a concentration of 1 mg/mL. Consequently, 1 mg/mL was identified as the most effective concentration for determining ALC. Finally, different quantities of PC-20 nanocomposite (1.0 μL–10.0 μL) were applied onto the GCE surface within BR buffer. The highest efficiency was achieved with 2.0 μL, reaching its peak response (Figure S2C); however, further increases in this quantity weakened the adherence of the modifier layer to the surface and impeded the diffusion of the analyte. As a result, the sensitivity of the modified GCE significantly decreased. Therefore, 2.0 μL was selected as the optimal composite volume.

### 3.4 pH Optimization

**Figure 12:**
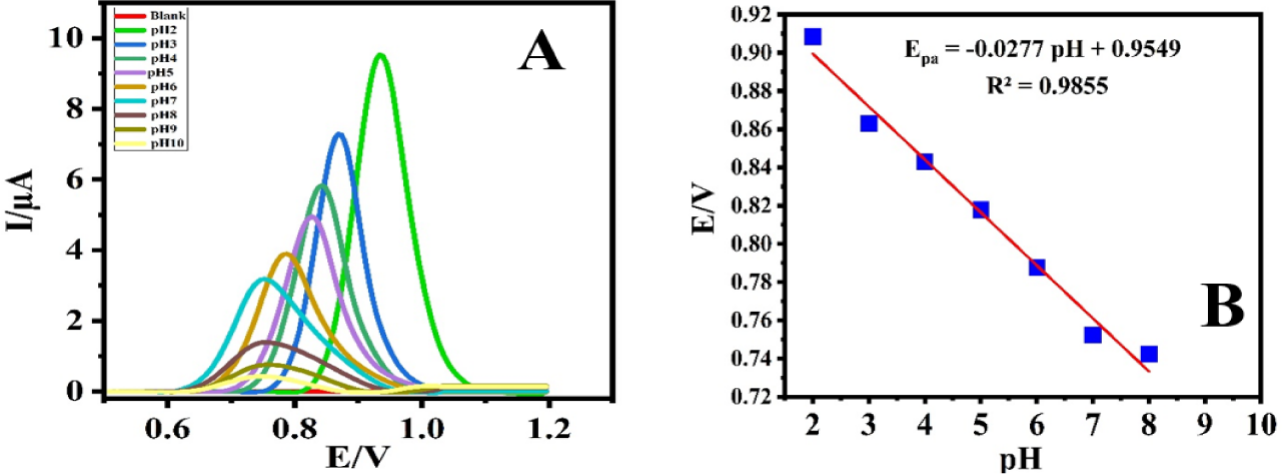
DPV of 0.1 mM ALC in BR on the surface of GCE/PC-20 at various pH levels (A), along with the correlation between peak potential (*E*_p_) and pH value (B).

The influence of pH on the determination of ALC (0.1 mM) using PC-20/GCE was additionally investigated through the DPV method, employing various pH levels (ranging from 2 to 10) of 0.1 M BR buffer (Figure 12A). As the pH value increased from 2 to 10, the oxidation peak curves shifted toward more negative potentials. This shift indicates the reduction of protons in the electrode reaction. ALC oxidation peak current observed reached its maximum at pH 2.0, establishing it as the optimal pH for entire experiments related to ALC detection. The slope of the potential–pH diagram (Figure 12B) was approximately 28 mV, which closely matched the theoretical slope of 29 mV, affirming that the electron’s number was double the proton’s number.^37^

### 3.5 Influence of the Scan Rate

**Figure 13:**
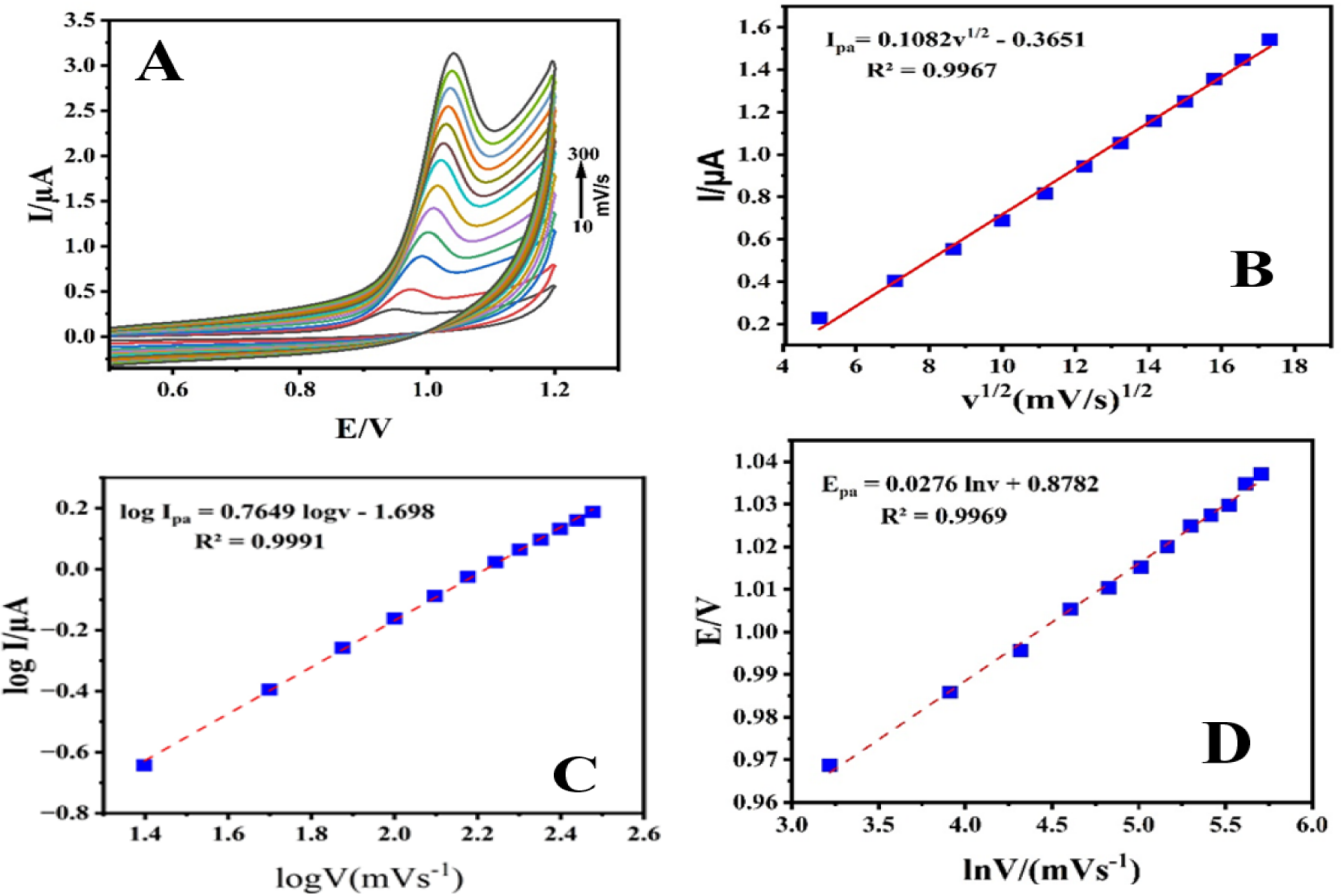
Cyclic voltammetry of 0.1 mM ALC in BR (pH = 2) on the surface of GGE modified with PC-20 at various scan rates (A), the correlation between *I*_pa_ vs υ^1/2^ (B), log *I*_pa_ versus log υ (C), and *E*_pa_ versus ln v (D).

The impact of potential scan rate (ν) on PC-20/GCE was studied by performing CV assay in a 10.0 μmol L^–1^ ALC solution in BR buffer (pH = 2.0), employing various scans 10–300 mV/s. As the scan rates increased, the irreversible oxidation peak currents of ALC showed a linear increase, as depicted in Figure 13A. In Figure 13B, a linear correlation between peak current and the square root of the scan rate was established using the following equation: *I*_pa_ (μA) = 0.1082 υ^1/2^ (V s^–1^) – 0.3651; (R^2^ = 0.9967). These findings suggest that the oxidoreduction reaction of ALC was controlled by a diffusion process.^38^

Additionally, a direct linear relationship was noted between the logarithm of *I*_p_ and the logarithm of υ, yielding a slope of 0.7649° (Figure 13C).

**Scheme 1:**
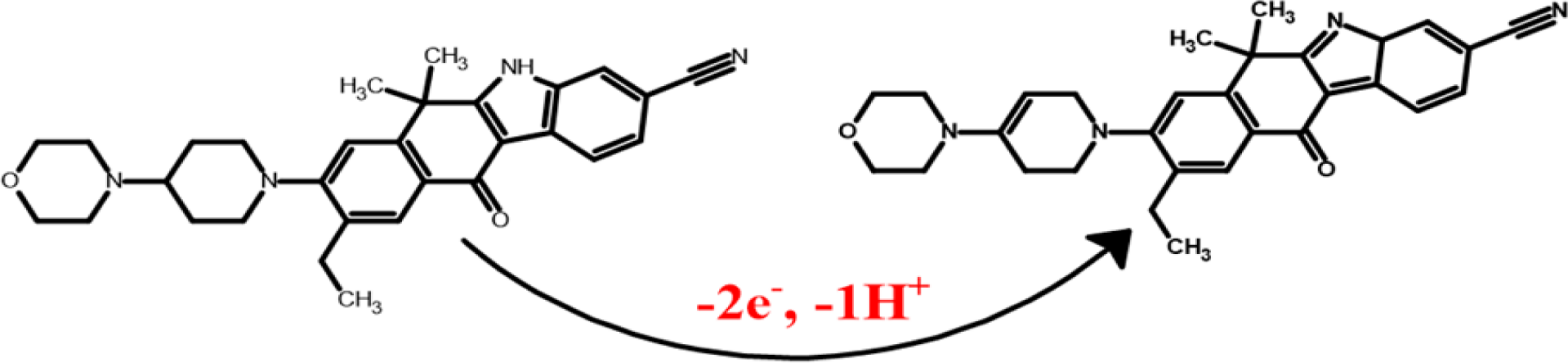
Potential Electro-Oxidation Mechanism of ALC

Finally, the correlation between the oxidation peak potential (*E*_pa_) and the natural logarithm of the scan rate (ln ν) was found to be linear, as described by the equation *E*_pa_ = 0.0276 ln (υ) + 0.8782, with a high degree of correlation (*R*^2^ = 0.9969) (Figure 13D). This relationship aligns with the Laviron equation.^39,40^ With an assumption of α being 0.5 to represent a completely irreversible electron transfer, the calculated value for *n* was approximately 1.86, although it was assumed to be 2. The irreversible oxidation process of ALC entails the transfer of two electrons and one proton, resulting in the formation of an oxidation product (Scheme 1).

### 3.6 Calibration

**Figure 14:**
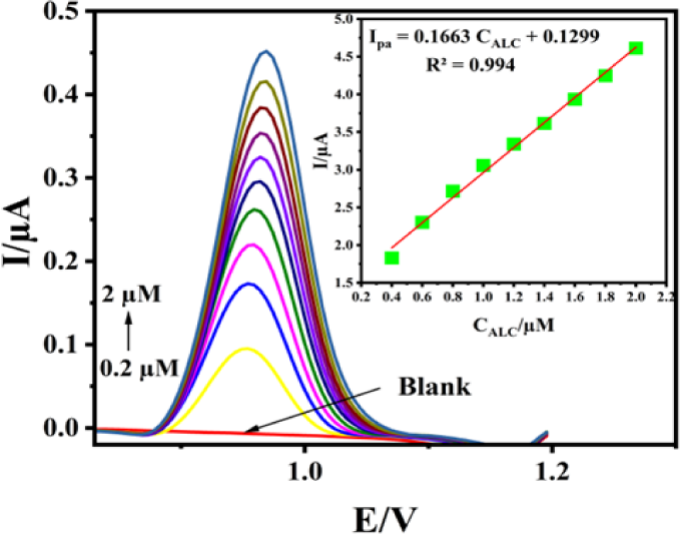
DPV of 0.2–2 μM ALC in BR solution at PC-20/GCE (A), and curve showing the correlation between *I*_pa_ and the concentration of ALC (B).

Using the optimized parameters, the constructed sensor was practically tested by employing ALC solutions across a concentration range of 0.2–2 μM, and calibration curves were generated through DPV measurements. As illustrated in Figure 14A, the oxidation peak currents of ALC displayed a notable increase corresponding to the rise in ALC concentration. Additionally, Figure 14B depicts a strong linear relationship between the concentration of *A* and its oxidation peak current. Furthermore, using the equation: LOD = 3S/m; LOQ = 10S/m (S = standard deviation of peak current, *m*= slope of the calibration curve). The detection limit (LOD) and quantification limit (LOQ) were established at 18.89 and 62.97 nM, respectively (Table 2).

**Table 2:** Performance Metrics Analysis of the Proposed Method^a^

Parameters	Value
Intercept	0.12994 ± 0.00636
Slope	0.1663 ± 0.00487
*n*	5
Determination coefficient (R^2^)	0.9940
Linear range (μM)	0.2–2.0
LOD (nM)	18.89
LOQ (nM)	62.97

a *n*: number of measurements.

### 3.7 Reproducibility, Repeatability, and Stability

To evaluate the repeatability of the PC-20/GCE, a set of 11 successive cycles was documented for 2 μM ALC, and the relative standard deviation (%RSD) was calculated (Figure S4A). The determined RSD was 2.33% for the 11 cycles suggesting outstanding repeatability of the PC-20/GCE.

Moreover, to assess the precision of the current sensor, the response of a 2 μM ALC was monitored across seven independently constructed modified electrodes, all fabricated under identical conditions. The resulting relative standard deviation (RSD) was 1.5%, demonstrating the excellent reproducibility of the employed method (Figure S4B).

The operational stability of the proposed sensor was assessed using the DPV method with an addition of 2 μM ALC. Notably, the peak current response showed a decrease of only 4% from the initial peak current value over 10 days (Figure S4C).

### 3.8 Selectivity

To assess the impact of commonly used excipients in pharmaceutical formulations, a series of studies were conducted, employing substances, such as paracetamol, ascorbic acid, l-arginine, l-methionine, potassium chloride, d-glucose, sodium sulfate, sodium nitrate, dopamine, and uric acid. Notably, significant interference was noted when the tolerance exceeded 5%. However, it was observed that even at concentrations 100 times higher than those typically encountered, these excipients did not disrupt the voltammetric signal of the ALC.

### 3.9 Real Sample Analysis

Finally, the electrochemical sensor PC-20/GCE was applied to detect ALC in both human urine and tablets, demonstrating the feasibility of the DPV method. The recoveries were determined using the standard addition method, as reported previously. Recoveries for ALC in human urine (spiked) and tablets ranged from 97.07% to 103.00%, with detailed results provided in Table 3. These findings reveal that the PC-20/GCE developed in this work can be used as an effective and reliable sensing platform for detecting ALC in biological fluids and pharmaceutical formulation.^41,42^

**Table 3:** Determination of ALC in Real Samples

Sample	Added (μM)	Found (μM)	RSD (%) (*n*=3)	Recovery (%)
Tablet	0.800	0.789	1.03	98.62
	1.400	1.359	2.10	97.07
	2.000	1.987	1.44	99.35
Human urine	0.800	0.824	2.05	103.00
	1.400	1.415	1.93	101.07
	2.000	2.040	2.63	102.00

## 4 Conclusion

In this study, we successfully synthesized a Co/Co_3_O_4_ heterostructure embedded in N-doped porous amorphous carbon and demonstrated its exceptional performance as an electrochemical sensor for the sensitive determination of ALC in various fluids. The synthesis process yielded a unique material with a synergistic combination of Co and Co_3_O_4_, which, when integrated into the N-doped carbon matrix, exhibited remarkable electrical conductivity, an enhanced surface area, and superior catalytic activity.

The electrochemical characterization of the sensor revealed high sensitivity and selectivity toward ALC, achieving a low detection limit and a wide linear range. The DPV method confirmed the sensor’s excellent operational stability, with only a minimal decrease in peak current response over repeated measurements, indicating its reliability for long-term use.

Moreover, the sensor’s performance in detecting ALC in real biological and pharmaceutical samples demonstrated its practical applicability. The robustness of the sensor, coupled with its ability to operate in complex fluid matrices without significant interference, underscores its potential for clinical and pharmaceutical applications.

Our findings highlight the effectiveness of the Co/Co_3_O_4_–N-doped porous carbon heterostructure as an advanced material for electrochemical sensing. This study paves the way for the further exploration and optimization of such heterostructures in the development of high-performance sensors for various analytes. The successful implementation of this sensor could significantly enhance therapeutic drug monitoring and contribute to more personalized and effective patient care.

Future research should focus on exploring the scalability of the synthesis process, the long-term stability of the sensor in various environments, and its performance with other pharmaceutical compounds.

## Data Availability

Data will be made available on request.

## Supporting Information Available

Additional experimental details, such as materials, reagents, and equipment used in the study; and important equations, an analysis of how concentration and the amount of the composite affect the results, and graphs from cyclic voltammetry and differential pulse voltammetry experiments (PDF)

## Notes

No approval of research ethics committees was required to accomplish the goals of this study because synthetic materials were used in the experimental study.
