# Supplementary material for: Synthesis of Co/Co3O4 Heterostructure in N-Doped Porous, Amorphous Carbon: A Superior Electrochemical Sensor for Sensitive Determination of Alectinib in Various Fluids
Source: ACS Omega. 2024 Oct 24;9(44):44282–92. doi: 10.1021/acsomega.4c04821 (PMC11541435; doi:10.1021/acsomega.4c04821)
Supplement: Supplementary file 1 — ao4c04821_si_001.pdf [file ao4c04821_si_001.pdf]

Supplementary Information

Synthesis of Co/Co_3o_4 Heterostructure in N-Doped Porous Amorphous Carbon: A Superior Electrochemical Sensor for Sensitive Determination of Alectinib in Various Fluids

Nesrin BUGDAY [a,*], Edoh Nicodème GABIAM[b],Nevin ERK[b,*], M.Soner BAY[b] , Asena Ayşe GENC[b] , Ozgur DUYGULU[c] , Sedat YAŞAR[a]

[a]İnönü Üniversity, Faculty of Science and Art, Department of Chemistry, 44280, Malatya,Turkey ,nbugday7@gmail.com;sedat.yasar@inonu.edu.tr

[b]Ankara University, Faculty of Pharmacy, Department of Analytical Chemistry, Ankara, Turkey; erk@pharmacy.ankara.edu.tr ; edohnicodemeg@gmail.com ; soner.bay@titck.gov.tr ; asenaaysegenc@gmail.com

[c]TUBITAK Marmara Research Center, Materials Technologies, 41470 Gebze, Kocaeli,

Turkey , ozgur.duygulu@tubitak.gov

Materials and Reagents

Benzimidazole (BIM), Cobalt acetate tetrahydrate ((CH3COO)2Co.4H2O) and ammonium hydroxide (NH3, 28–30% aqueous solution) were purchased from Alfa Aesar. Sodium chloride (NaCl), and potassium chlorate (KIO3) Sigma-Aldrich. All chemicals were analytical reagents and used as received without any further purification. Aqueous solutions were prepared with ultrapure water (>18 MΩ cm-1).

Glucose (99.5 %), L-arginine (98.0 %), L-methionine, sodium hydroxide, potassium hexacyanoferrate (III) ($K_3Fe(CN)_6$, 99.5 %), hydrochloric acid, sodium Acetate, ascorbic acid, uric acid (99.0 %), acetic acid, potassium chloride, sodium phosphate, sodium sulfate, potassium chloride, sodium sulfate, were purchased from Sigma Aldrich Co. (https://www.sigmaaldrich.com, Germany). Britton-Robinson buffer was made of boric acid, phosphoric acid, potassium chloride, and acetic acid solutions. The stock solution of ALP was prepared in acetonitrile: water (1:1). Human plasma samples were also acquired from Sera-Flex Inc. All chemical compounds were analytical grade and used without additional refinement.

Apparatus

XRD analysis of the prepared materials was carried outusing Rigaku Rint 2000 X-ray Diffractometer between 2 and 80 /min with a scan rate of 2/min.

The specimens were investigated by JEOL JSM 6510LV scanning electron microscope (SEM) at 15 kV. The morphology at nanoscale was further observed by JEOL JEM 2100 High Resolution Transmission Electron Microscope (LaB6 filament) operated at 200 kV and equipped with an Oxford Instruments X-Max 80T Energy Dispersive Spectrometer (EDS) system. Carbon support film coated copper TEM grids (Electron Microscopy Sciences, CF200-Cu, 200 mesh) were used. Images were taken by Gatan Model 833 Orius SC200D CCD Camera. HRTEM images were taken by by Gatan Model 794 Slow Scan CCD Camera. Gatan Microscopy Suite (GMS) 2 software was used. For diffraction pattern indexing CrystBox software was used [M. Klinger. CrysTBox - Crystallographic Toolbox. Institute of Physics of the Czech Academy of Sciences, Prague, 2015. ISBN 978-80-905962-3-8. URL http://www.fzu.cz/~klinger/ crystbox.pdf]. The elemental composition and phase structure were analyzed by X-ray photoelectron spectroscopy (XPS) were recorded using a Specs-Flex XPS instrument in the range of 200-4000 eV.

Voltametric experiments were carried out using AUTO LAB system with PGSTAT204 electrochemical workstation (Metrohm Inc., Switzerland) with a glassy carbon electrode system in a one-compartment of 10 mL electrochemical cell. All electrochemical measurements were performed at 25 °C unless otherwise specified.

The Randles-Sevcik equation: $I = (2.69 \times 10^5)\, n^{\frac{3}{2}}\, A\, D^{\frac{1}{2}}\, v^{\frac{1}{2}}\, C_0 \quad (S1)$

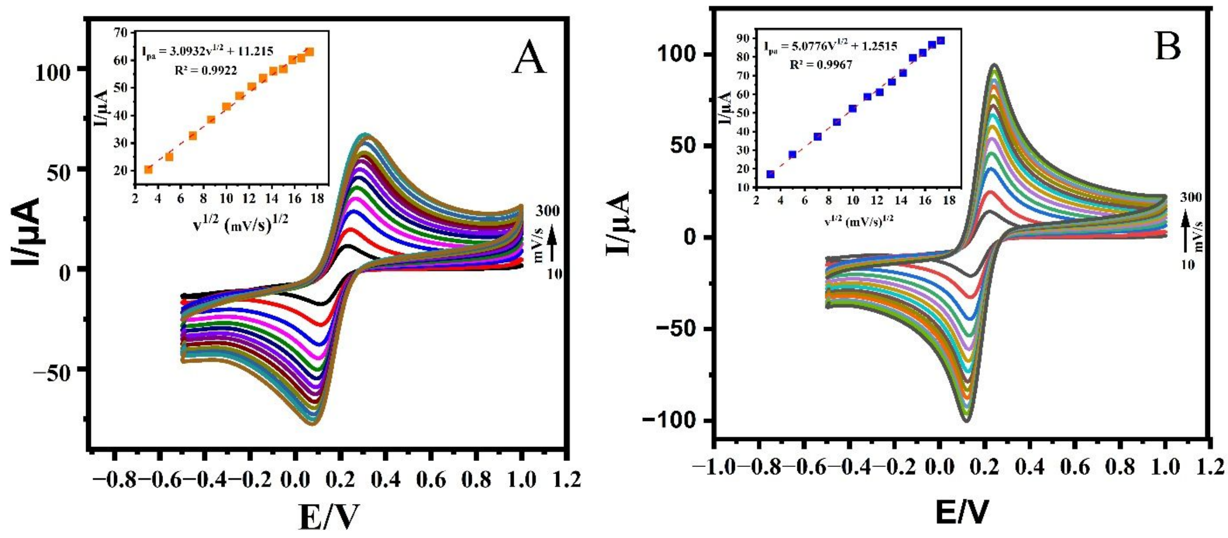

Figure S1. The recorded CV curves at various scan rates in the presence of 5.0 mM [Fe (CN) 6]$^{3-/4-}$ containing 0.1 M KCl on (A); bare GCE, (B) and PC-20/GCE

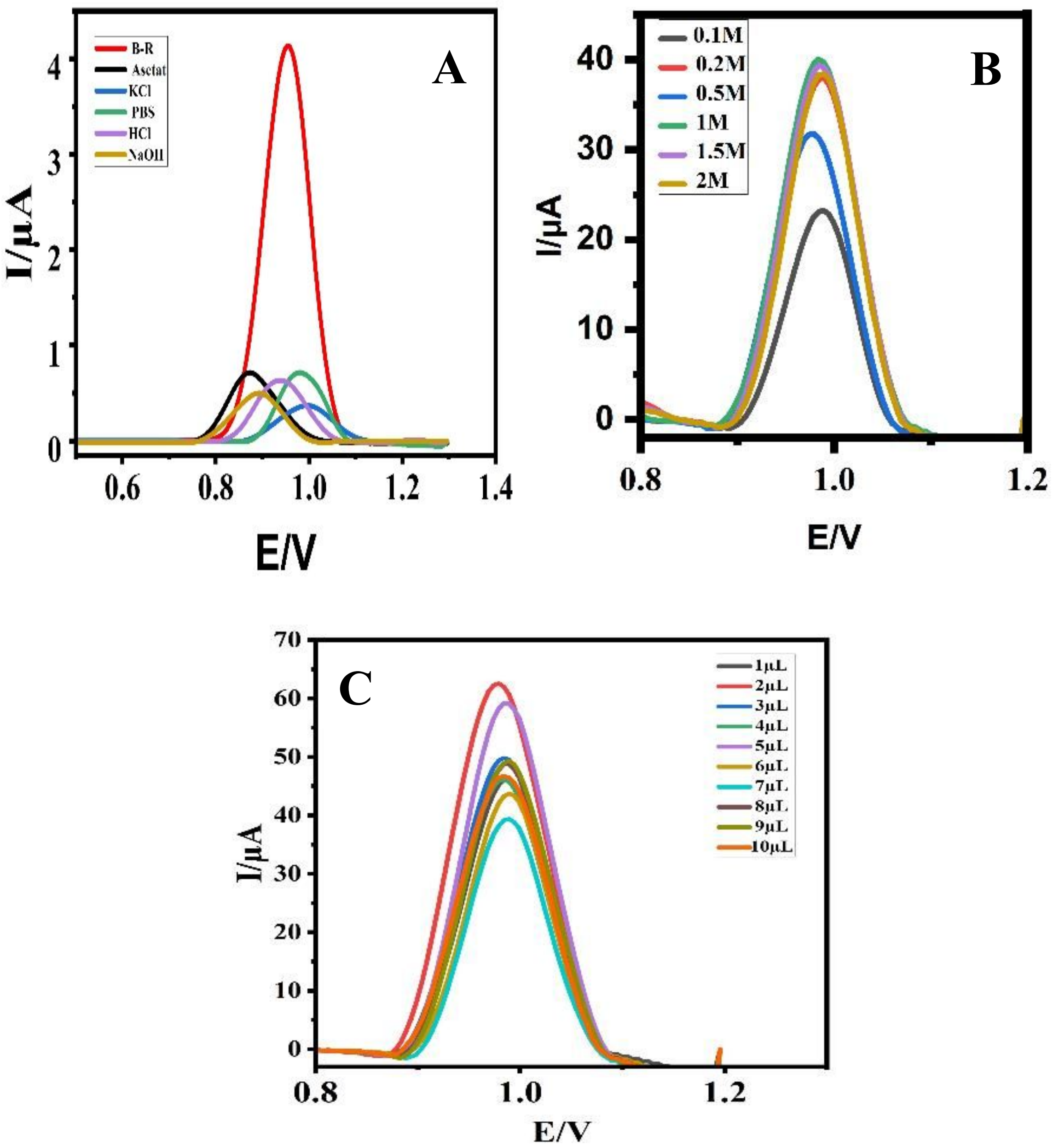

Fig. S2. Influence of supporting electrolyte (A), the concentration (B), and the amount (C) of PC-20 composite on the oxidation peak currents of 0.1 mM ALC

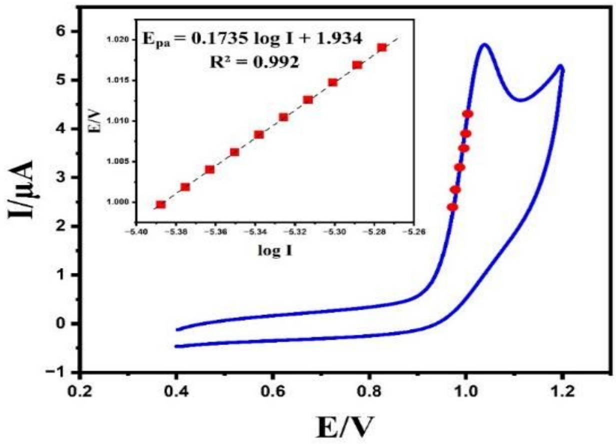

Fig. S3. Tafel plot for of 0.1mM ALC with scan rates of 100 mV s^{-1} on the surface of PC-20/GCE.

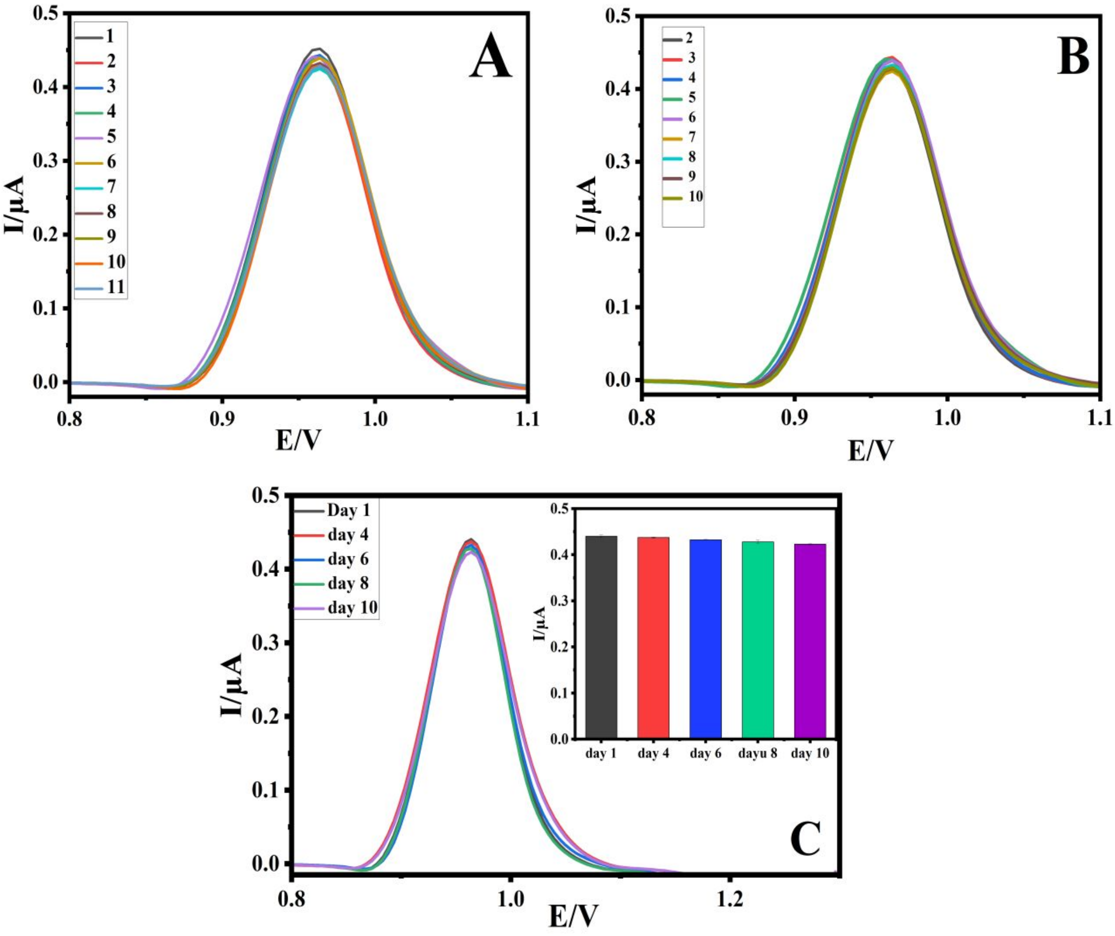

Fig. S4. Repeatability (A), reproducibility (B) and stability of 2.0 μM ALC at PC-20/GCE in B-R buffer (pH 2.0).

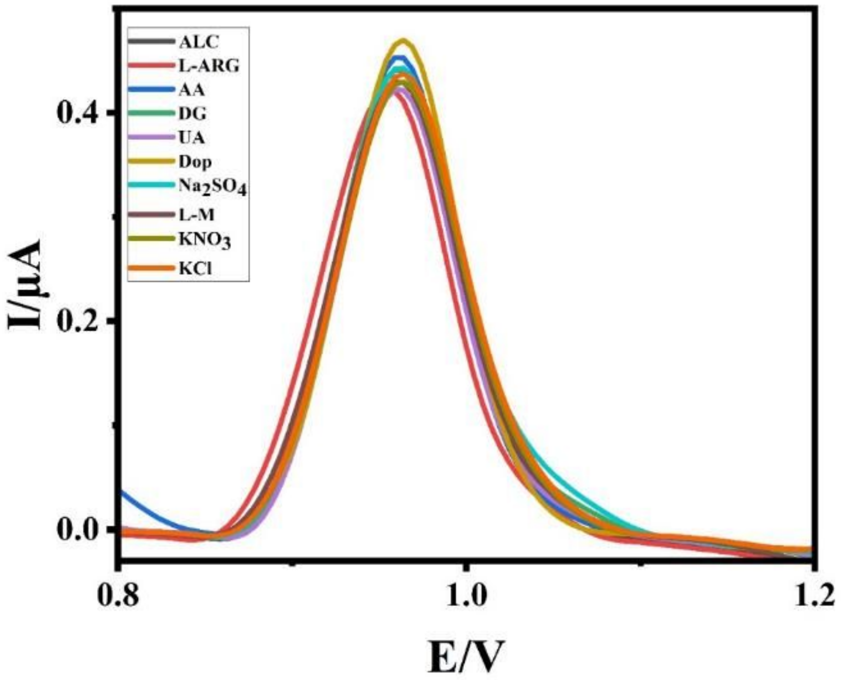

Fig. S5. Selectivity of 2.0 μM Alectinib at PC-20/GCE in B-R buffer (pH 2.0).
